# Whipped chickpea aquafaba as a fat replacer in ice cream: Effect on sensory and physicochemical properties

**DOI:** 10.1111/1750-3841.17605

**Published:** 2024-12-15

**Authors:** Andrea Balivo, Giulia d'Errico, Alessandro Genovese

**Affiliations:** ^1^ Department of Agricultural Sciences University of Naples Federico II, Piazza Carlo di Borbone 1 Portici Naples Italy

**Keywords:** dairy cream replacement, fat reduction, ice cream texture, SPME‐GC/MS, whipped chickpea aquafaba

## Abstract

**Abstract:**

Whipped chickpea aquafaba (WAF) exhibits foaming, emulsifying, and gelling properties, making it a potential ingredient for replacing cream in ice cream formulations. The aim of this study was to use WAF in combination with whey protein isolate (WPI) to produce low‐fat coffee ice cream with 50% (L50) and 80% (L80) milk cream replacement. The melting rate, color, texture, volatile compounds, and sensory attributes were analyzed to explore the physical, chemical, and sensory properties of the low‐fat ice creams compared to their full‐fat counterparts. Fat substitute performance varied based on cream replacement levels, with L50 1:0 (WAF:WPI) and L80 1:0 achieving 25% and 40% reductions in calorie content, respectively. The foaming properties of aquafaba resulted in an increased overrun, particularly in formulations where the cream reduction was 50%. While the 80% replacement showed higher intensity of “hardness,” “brown color,” and “melting” sensory descriptors, the 50% cream‐replaced ice cream showed only slight differences compared to the full‐fat version, like a higher perception of ice crystals. The combination of WAF and WPI in ice cream provides sensory properties and releases pyrazines, responsible for the coffee aroma, similar to full‐fat ice cream. The 1:1 WAF:WPI mixture for 50% cream reduction provided a suitable cream replacement, as this formulation resulted in ice cream that was not identified as different from the full‐fat control ice cream in the triangle test.

**Practical Application:**

This study demonstrates that whipped aquafaba, a by‐product of chickpea processing, can be effectively used to reduce the fat content in ice cream without compromising sensory quality. In addition to promoting the development of healthier low‐fat ice creams, this approach also contributes to food industry sustainability by reusing a commonly discarded by‐product.

## 1 INTRODUCTION

Ice cream is a frozen dairy dessert consumed worldwide by people of all ages. Concerns about health and nutrition have led to the emergence of low‐fat ice cream as a healthier alternative (Genovese et al., 2022). The high fat content of ice cream is associated with an increased risk of obesity, including childhood obesity, and related health problems, such as hypertension, diabetes, and cardiovascular diseases (Krystyjan et al., 2015). Scientific opinions recommend that the intake of saturated fatty acids in the human diet should not exceed 10% of the daily energy intake, as well as the dietary cholesterol intake should not cover >300 mg per day (EFSA, 2017; WHO, 2019). Consumers have become more conscious of the health problems related to high‐fat and high‐calorie diets and are looking for food products with a reduced fat content to improve their psychophysical well‐being (Küster et al., 2022).

Milk cream accounts for approximately 10%–18% of the ice cream formulation, depending on the ice cream product category, and plays a crucial role in determining the physical and texture properties as well as the kinetics of aroma release (Ayed et al., 2018; Genovese et al., 2022). Reducing the fat content of ice cream continues to be a focus of current research and industry, with the aim of producing low‐fat ice cream with sensory and technological characteristics similar to conventional full‐fat ice cream. Many studies have been published on the effect of different fat replacers in ice cream on its physical, chemical, and sensory properties. Whey proteins are among the most investigated fat substitutes and have shown promising results in terms of aroma retention and sensory properties when used as a partial fat replacer in ice cream (Feyzi et al., 2020; Yilsay et al., 2006). On the other hand, the use of carbohydrate‐based fat replacers such as inulin, plant gums, and other hydrocolloids can help improve the texture and thermal properties of ice cream (Hashemi et al., 2015; Javidi et al., 2016; Karaca et al., 2009). Other ice cream reformulation strategies consider the inclusion of plant‐based ingredients derived from food industry by‐products to promote greater environmental sustainability (Anwar et al., 2022; da Silva et al., 2020; Kasapoglu et al., 2023; Pontonio et al., 2022). Despite progress, previous reviews on the topic have highlighted that a combination of different types of fat replacers may ensure an optimal formulation of low‐fat ice cream in terms of physical, chemical, and sensory quality (Akbari et al., 2019; Genovese et al., 2022).

Aquafaba is a viscous liquid drained from cooked or canned pulses, usually chickpeas, which consists of water (∼95%), carbohydrates (i.e., sugars, soluble, and insoluble fiber), low molecular weight proteins (∼1%; ≤ 24 kDa), saponins, and some Maillard reaction products (Erem, Icyer, et al., 2023; He et al., 2021). Scientific research on the use of aquafaba, primarily as a replacer for egg or animal protein, is <5 years old, despite the vegan community's interest in reusing the cooking water of pulses, which dates back almost a decade (Roessel, 2014). Chickpea aquafaba exhibits emulsifying, foaming, and gelling properties, enabling the formation of stable gels, although these properties are influenced by factors such as legume composition, genotype, cooking time, pressure, temperature, and the ratio of water to seeds (de Barros Miranda et al., 2024; Stasiak et al., 2023). Aquafaba presents an opportunity for reducing the environmental load by recycling by‐products into value‐added food ingredients (Yazici et al., 2022).

The emulsifying, gelling, and foaming properties of aquafaba have led to its use as an egg substitute in bakery products, meringue, and mayonnaise to mimic the foaming and emulsifying properties of eggs (Lafarga et al., 2019; Meurer et al., 2020; Mustafa et al., 2018; Serventi et al., 2018). Previously, aquafaba has been used to replace palm oil in pound cakes at levels of up to 35%, resulting in cakes with a higher specific volume (Karatay et al., 2022). Recently, aquafaba was added to a fruit‐based frozen vegan dessert, showing promising improvements in characteristics such as overrun, sensory perception, and rheological properties, although the differences are discussed mainly in relation to the variation in the sucrose‐to‐fruit ratio in the samples (Erem, Akdeniz, et al., 2023).

The functional properties of aquafaba have led us to hypothesize its potential use as a fat substitute, particularly as a replacer for cream in ice cream. Since there is still a need to find a fat substitute capable of adequately emulating the functional properties of cream in ice cream, the aim of this study was to partially replace the cream in ice cream (by 50% and 80%) using a combination of whipped chickpea aquafaba and whey protein isolate (WPI) to develop a low‐fat ice cream that closely mimics the conventional full‐fat counterpart. The sensory and physical properties, as well as the release of volatile compounds, were examined in the low‐fat ice cream formulations in comparison to the full‐fat ice cream.

To the best of our knowledge, this is the first study exploring the use of whipped aquafaba (WAF) as a fat replacer in ice cream. These findings may help ice cream producers to develop healthier and more acceptable low‐fat ice creams for the consumer, as well as offer the opportunity to contribute to the virtuous reuse of by‐products of the food industry.

## 2 MATERIALS AND METHODS

### 2.1 Ice cream formulation and production

Eleven formulations of ice cream (including control full‐fat ice cream) were prepared. The formulations of low‐fat ice creams (L) were based on two factors: the degree of cream replacement (50% and 80%) and the fat replacement ingredients used, that is, WAF, WPI, and their combinations, as indicated in Table 1. Samples 1:0 and 0:1 in Table 1 represent ice creams produced exclusively with WAF or WPI, respectively, while the intermediate formulations indicate blends of both ingredients used to replace milk cream. The replacement of cream with 100% WPI was chosen as it is commonly used in the development of low‐fat ice creams and to better investigate the effect of aquafaba in combination with different levels of WPI.

**TABLE 1:** Formulation of coffee ice cream containing different levels of milk cream, whipped aquafaba and whey protein isolate.

			Ingredient (wt%)								
Sample code	Fat replacement level (%)	Fat replacer composition	Water	Skimmed milk powder	Sucrose	Milk cream (36% fat)	Instant coffee	Stabilizer	Emulsifier	WPI	WAF
Full‐fat	0	–	51.1	5.7	13.7	27.5	1.6	0.2	0.2	0	0
L50 1:0	50	100% WAF	51.1	5.7	13.7	13.7	1.6	0.2	0.2	0	13.7
L50 3:1	50	75% WAF 25% WPI	51.1	5.6	13.7	13.7	1.6	0.2	0.2	3.4	10.3
L50 1:1	50	50 % WAF 50% WPI	51.1	5.6	13.7	13.7	1.6	0.2	0.2	6.9	6.9
L50 1:3	50	25% WAF 75% WPI	51.1	5.6	13.7	13.7	1.6	0.2	0.2	10.3	3.4
L50 0:1	50	100% WPI	51.1	5.7	13.7	13.7	1.6	0.2	0.2	13.7	0
L80 1:0	80	100% WAF	51.1	5.7	13.7	5.5	1.6	0.2	0.2	0	22.0
L80 3:1	80	75% WAF 25% WPI	51.1	5.6	13.7	5.5	1.6	0.2	0.2	5.5	16.5
L80 1:1	80	50 % WAF 50% WPI	51.1	5.6	13.7	5.5	1.6	0.2	0.2	11.0	11.0
L80 1:3	80	25% WAF 75% WPI	51.1	5.6	13.7	5.5	1.6	0.2	0.2	16.5	5.5
L80 0:1	80	100% WPI	51.1	5.6	13.7	5.5	1.6	0.2	0.2	22.0	0

Abbreiations: WAF, whipped aquafaba; WPI, whey protein isolate.

For the preparation of ice creams, water (51.1 wt%), milk cream at 36% fat (27.5 wt%, in full‐fat control sample), sucrose (13.7 wt%), skimmed milk powder (5.7 wt%; Bongiovanni S.p.a.), soluble coffee (1.6 wt%), emulsifier (mono‐ and diglycerides of fatty acids; 0.2 wt%; SaporePuro, Gioia Group s.r.l.), and stabilizer (mixture of locust bean gum, guar gum, sodium alginate, and agar agar; 0.2 wt%; Special Ingredients) were mixed together using a stirring device at a temperature of 60°C on an induction heating plate. Soluble coffee was chosen to mask the legume‐like aroma of aquafaba, as observed in preliminary trials with unflavored ice cream, as well as for its ease of dosing and low caloric impact. For the low‐fat formulations, cream was replaced by 50% and 80% with fat replacers, as detailed in Table 1. The ice cream mix was homogenized using an Ultra Turrax T25 homogenizer (IKA Instruments, Staufen, Germany) at 20,500 rpm for 15 min, aged at 4°C for 24 h and transferred into the ice cream maker (Springlane GmbH), which operated for 50 min, gradually decreasing the temperature to reach −23°C. Hardening continued in the ice cream maker for 50 min at −35°C.

The liquid ingredients were initially placed in the mixing bowl, and when the temperature reached 60°C, the dry ingredients were gradually added while continuing to stir to ensure proper incorporation and uniform dispersion of the mix. Chickpea aquafaba was whipped using an electric planetary mixer for 5 min at a power of 175 watts before being incorporated into the cooled ice cream mix, ensuring uniform distribution. The aquafaba of the present experiment was obtained from the cooking liquid of canned chickpeas from the same production lot, purchased in a local supermarket. The ice creams were produced with food‐grade ingredients and packaged into 1000 mL polystyrene pans (model Gelato YETI XXL, Alcas s.p.A.), then stored in a freezer at −20°C for 4 weeks before analyses. Each ice cream product was replicated twice. Before freezing, the overrun (%), that is, the incorporation of air into the ice cream, was calculated per sample by comparing the weight of ice cream mixture and ice cream in a fixed volume container using the following equation (Akbari et al., 2016):

$$\mathrm{Overrun}\;\;\left(\%\right)=\frac{W_{\mathrm{ICM}}-W_{\mathrm{IC}}}{W_{\mathrm{IC}}}\times 100$$

where W_ICM_ is the weight of the ice cream mix and W_IC_ is the weight of the final ice cream.

### 2.2 Sensory analysis

Ice cream samples were analyzed for sensory properties using quantitative descriptive analysis (QDA) methodology. A panel of 12 trained assessors, aged between 25 and 50 and recruited from the Department of Agricultural Sciences at the University of Naples Federico II, conducted the sensory analysis tests. The procedure was based on the guidelines laid down in the Declaration of Helsinki for studies on human subjects. All the assessors had studied the protocol and provided their written informed consent before the experiment took place. The panel members generated the list of descriptors by tasting samples in a group discussion, choosing the most relevant attributes for evaluating the sensory characteristics of ice cream. The descriptors for each major sensory attribute category included brown color and shape retention for appearance; melting time, hardness, sandy texture, and ice crystals for texture; sweetness and bitterness for taste; and milk cream, caramel, and coffee for aroma (Table 2).

**TABLE 2:** Attributes generated and evaluated by the panel in the sensory analysis of the ice creams and their definition.

Descriptor	Definition
**Visual descriptors**	
Brown color	Light brown to dark brown under white light.
Density/shape	Degree of shape retention and compactness.
**Texture descriptors**	
Melting time	Time (seconds) required to melt a spoonful of ice cream in the mouth.
Hardness	Force required for the first compression of the sample between the tongue and the palate. The higher the force, the higher the hardness.
Sandiness	Perception of a gritty sensation during consumption, reminiscent of fine grains or sand‐like particles in the mouth.
Ice crystal	The immediate perception of crystal‐like particles within the sample. This measurement is taken immediately after sample has been placed in the mouth. The crystals often dissolve quickly at first manipulation.
**Aroma descriptors**	
Milk cream persistence	The duration and intensity of a milk cream perception after the ice cream is removed from the mouth.
Caramel persistence	The duration and intensity of a caramel perception after the ice cream is removed from the mouth.
Coffee persistence	The duration and intensity of a coffee perception after the ice cream is removed from the mouth.
**Taste descriptors**	
Sweetness	Fundamental taste imparted by sucrose.
Bitterness	Fundamental taste imparted by caffeine or quinine.

The intensity of each attribute was quantified using a structured scale ranging from 0 (no perception) to 10 (very intense perception). A complete randomized block design was implemented for all tests conducted in the five analysis sessions. The 11 ice cream samples were divided among the five sessions, presenting in each session the full‐fat control ice cream and two experimental samples in a randomized product presentation order.

Two triangle tests (ISO, 2007) were conducted on the 50% and 80% reduced fat ice cream samples that were most similar to the full‐fat control ice cream. The aim was to verify if the low‐fat ice creams that were most similar to the control could be discriminated by the panel members. In the first triangle test, a comparison was made between full‐fat ice cream and a 50% fat‐replacement ice cream that exhibited greater similarity to the full‐fat counterpart. Similarly, a second triangle test was conducted for the 80% fat‐replacement ice cream. In each test, three samples, two of which were identical, were presented simultaneously to the panelists, in two randomized serving orders for each comparison. The panelists needed to identify the odd sample using the sense of smell, taste and touch.

For sensory tests, 30 g of ice cream sample, coded with blind three‐digit numbers, were evaluated at 20°C. All ice cream samples were analyzed after 1 month of storage at −20°C.

### 2.3 Melting rate

The melting rate was measured in triplicate for each sample at 25°C after 1 month of frozen storage. A 50 g rectangular block of ice cream, initially at −20°C, was cut from the center of the pan and placed on a 0.2 cm wire mesh screen over a funnel to melt (Tiwari et al., 2015). Every 5 min, for 1 h, the weight of melted ice cream collected below in a beaker was recorded. The results were calculated using the following equation (Guler‐Akin et al., 2021).

$$\mathrm{Melting}\;\mathrm{rate}\;\;\left(\%\right)=\frac{W_{\mathrm{MIC}}}{W_{\mathrm{IC}}}\;\times 100$$

where W_MIC_ is the weight of melted ice cream and W_IC_ is the weight of ice cream.

### 2.4 Texture and color analysis

The hardness of the ice creams was evaluated using a Texture Analyzer FRTS‐50N (IMADA) equipped with a 50 N load cell according to Guler‐Akin et al. (2021) method with slight modifications. A quantity of approximately 50 g of ice cream was placed in a cup, carefully forming a uniform ice cream block with a flat surface and with a height and diameter of about 4 cm. The sample was compressed, in a single compression cycle, to 20% of its original height with a test speed of 1 mm s^−1^ and a trigger force of 5 g, using a cylindrical probe with a diameter of 5 mm. The hardness (N) was obtained from the maximum peak force of the force‐time curve (Force Recorder Professional Software, version 1.03, IMADA). Measurements of five replicates by type of ice cream were carried out at room temperature of 20°C, after the ice cream was tempered overnight at −13°C. The analyses were performed after 1 month of storage at −20°C.

The color of ice creams was measured with a portable color meter FRU^®^ WR‐10QC (Shenzhen Wave Optoelectronics Technology Co Ltd) equipped with a photodiode array sensor with a sensor head of 8 mm in diameter. The color was measured in five replicates on the surface of each sample to determine CIELAB L*, a*, and b* color parameters of ice creams. The intensity of L*, a*, and b* values indicate whiteness (100)/darkness (0), redness (+ values)/greenness (− values), and yellowness (+ values)/blueness (− values), respectively. ΔE values were determined using the following equation:

$$\Delta E=\sqrt{{\left(L-L_{r}\right)}^{2}}+{\left(a-a_{r}\right)}^{2}+{\left(b-b_{r}\right)}^{2}$$

where the subscript ‘*r*’ indicates the CIELAB values of the full‐fat reference sample.

### 2.5 Extraction and analysis of volatile organic compounds

The extraction of volatile organic compounds (VOCs) was performed using headspace solid‐phase microextraction (SPME) technique according to the procedure described by Balthazar et al. (2018) with minor adaptations. The ice cream was sampled from the center of the original container, excluding the upper 1 cm layer, utilizing a syringe. Consequently, 2.25 g of ice cream and 2.25 g of saturated NaCl solution were added into a 15 mL dark glass vial. The vial was maintained at a constant temperature of 40°C for 20 min. The 2‐cm length stationary phase SPME fiber (Supelco Co., Bellefonte), equipped with a 50/30 µm thick divinyl‐benzene/carboxen/polydimethylsiloxane (DVB/CAR/PDMS), was inserted through the Teflon septum in the vial and exposed to sample headspace for 30 min at 40°C.

The VOCs were desorbed directly in the injector port of gas chromatograph (GC) kept at a temperature of 250°C in split mode with a 4:1 split ratio for 10 min. Volatile compound analysis was performed on an Agilent 7890A GC System GC coupled to an Agilent 5975C VL MSD with Triple‐Axis‐Detector mass spectrometer (Agilent Technologies, Inc.). GC was equipped with a Zebron ZB‐WAX capillary column (60 m × 0.25 mm i.d. × 0.25 µm film thickness 100% polyethylene glycol). Helium was employed as the carrier gas at a flow of 1 mL/min. The temperature program was 40°C for 10 min, followed by a ramp of 5°C/min up to 240°C with an 11‐min hold period at the final temperature (Balivo, Sacchi, et al., 2024). Compounds were detected by mass spectrometry in the electron impact ionization mode at 70 eV in the range *m*/*z* 33–350. The identification of compounds was performed by comparing retention times and mass spectra obtained by analyzing pure reference compounds in the same conditions. All chemical standards were supplied by Sigma‐Aldrich. In addition, the identification was confirmed by comparing mass spectra with those of the National Institute of Standards and Technology database. The source temperature of MS was 230°C, the quadrupole temperature was 150°C, and the interface temperature was 250°C.

For quantitative purposes, spectra were recorded in the selective ion monitoring mode. The selected ions monitored were listed in Table 3. Quantification was performed by integrating the peaks of each compound. Peak area data were processed by MSD ChemStation 5975 TAD Data Analysis software (Agilent Technologies).

**TABLE 3:** Volatile compounds, odor descriptors, odor threshold and ion fragments monitored in ice cream samples.

Compound	Ion fragments (*m*/*z*)	Odor threshold (µg·L^−1^)	Log P_OW_	Odor descriptors
2‐Methylpyrazine	94‐67	60	0.49	Nutty, cocoa, roasted^a^
2,5‐Dimethylpyrazine	108‐107	80	1.03	Hazelnut, roasted, cocoa^ab^
2,6‐Dimethylpyrazine	108‐107	9,000	1.03	Coffee, nutty^b^
Ethylpyrazine	108‐107	6,000–22,000	0.98	Nutty, peanut, coffee, meaty^ab^
2‐Ethyl‐6‐methylpyrazine	121‐122‐58	40	1.53	Peanut, roasted^a^
2‐Ethyl‐5‐methylpyrazine	121‐122‐58	100	1.53	Nutty, roasted^a^
2,3,5‐Trimethylpyrazine	122,81	9	1.58	Roasted, nutty^a^
2‐Methylbutanal	58‐71	1.3	1.23	Musty, chocolate, nutty^b^
3‐Methylbutanal	58‐71	0.35	1.23	Malty, nutty, chocolate^b^
2,3‐Butanedione	57‐100	30	−0.85	Buttery, dairy, creamy^ac^
2‐Hydroxy‐3‐methyl‐2‐cyclopenten‐1‐one	112‐83	300	1.29	Sweet aromatic, caramel^a^
Guaiacol	109‐124	2.5	1.34	Phenolic, smoky, burnt^ac^
4‐Vinylguaiacol	107‐135‐150	0.75	2.24	Phenolic, clove, spicy^ad^
2‐Acetylthiazole	112‐127	10	0.67	Roasty, popcorn^c^

*Note*: Log P_OW_ indicates the logarithm of the partition coefficient of a compound between octanol and water. Predominantly hydrophilic molecules have negative log P_OW_ values, while positive values indicate increased hydrophobicity.

The odor threshold values were taken from Leffingwell & Associates (calculated in water). a = Ayseli et al. (2021); b = Tagliamonte et al. (2023); c = Culleré et al. (2013); d = Qian et al. (2002).

As the aim of the study was to compare the relative levels of volatile compounds released in the vapor phase in different ice cream samples containing the same amount of coffee aroma, no real quantification was required (Ayed et al., 2018). Therefore, the amount of each volatile compound in the ice cream was reported as absolute peak areas and expressed as a percentage with respect to the control full‐fat ice cream sample, which was set at 100%. The use of absolute peak area allows the measurement of the effect of different matrices on each volatile compound without the addition of errors due to the effect of the matrix on the internal standards. The efficiency of the SPME fiber was determined to ensure that the absolute peak area was not affected by fiber wear, as the GC/MS analysis was carried out over 3 weeks with approximately eight runs per day, 3 days per week. Extraction efficiency was determined by repeating the analyses on the same sample of ice cream on a daily basis with the addition of 20 µL of 2‐methyl‐3‐heptanone as an internal standard (12.5 mg/L, 99%, Sigma‐Aldrich). The test calculated an average loss of fiber efficiency of approximately 20% from the first week to the second week and a further loss of 7% in the third week. Correct assessment of fiber efficiency was demonstrated by the semi‐quantitative data using the added internal standard, which showed an average coefficient of variation for all volatiles of around 7%. For this reason, all data presented have been normalized on the basis of the extraction efficiency measurement. A new fiber was conditioned at 270°C for 1.5 h before starting the SPME‐GC/MS analysis. A blank test was performed before each analysis. Three replicates per sample were made.

### 2.6 Statistical analysis

Results were expressed as the mean ± standard deviation. Statistical analysis and visualization were carried out in XLStat environment (Version 2019 v.2.2), an add‐in software package for Microsoft Excel (Addinsoft Corp.). Differences in instrumental and sensory variables between full‐fat ice cream and experimental low‐fat ice cream samples were assessed by analysis of variance with Tukey's HSD test, for a significance level set at *p* ≤ 0.05. Partial least squares regression (PLSR) analysis was performed to investigate the correlativity between sensory and instrumental data and to intercept the ice cream samples most similar to full‐fat ice cream. To assess the differences in the triangle tests, data were analyzed by counting the number of correct responses (correctly identified “different” sample) and the number of total responses. These numbers were compared with critical values as described by Meilgaard et al. (1999) to determine significant differences.

## 3 RESULTS AND DISCUSSION

### 3.1 Sensory analysis

**FIGURE 1:**
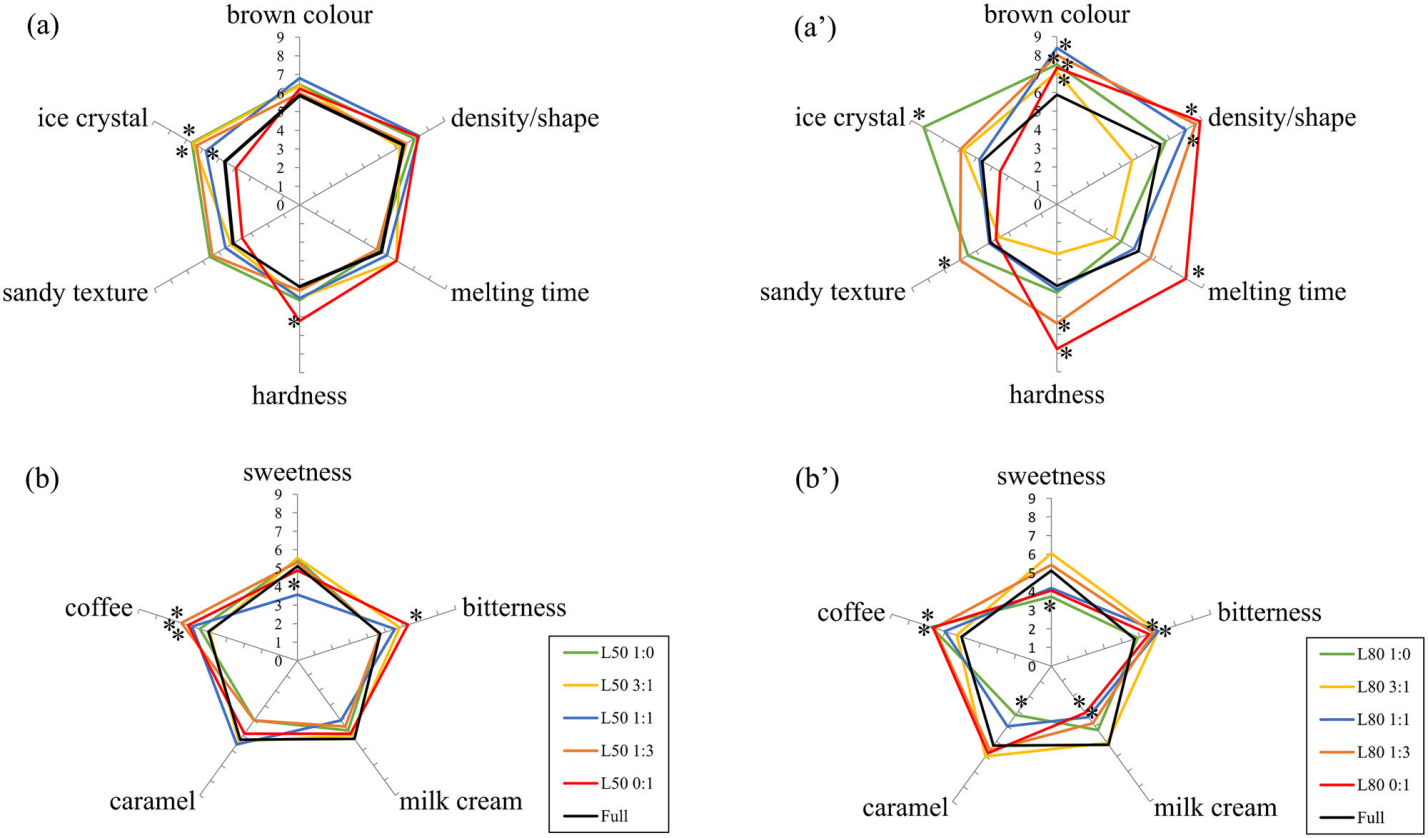
Sensory quantitative descriptive analysis (QDA) profile of visual, texture (a and aʹ), taste and aroma (b and bʹ) descriptors perceived in 50% and 80% cream replacement ice cream samples. The asterisk indicates significant differences (*p* < 0.05) compared to the full‐fat control ice cream. See Table 1 for sample code details.

The sensory properties of low‐fat coffee ice cream samples (L) were evaluated and compared to those of the control full‐fat ice cream. The mean intensity scores of the sensory evaluation of the ice cream samples in which the fat was replaced by 50% (L50, left side of the figure) and 80% (L80, right side of the figure) are shown in Figure 1. In particular, the upper part of figure shows the results regarding appearance and texture descriptors for 50% fat‐reduced ice cream (Figure 1a) and 80% fat‐reduced ice cream (Figure 1aʹ), while the lower part shows the results of taste and aroma descriptors for 50% fat‐reduced ice cream (Figure 1b) and 80% fat‐reduced ice cream (Figure 1bʹ).

The sensory response to the ice creams was affected by the reduction in milk cream content. Notably, greater differences emerged in L80 than L50 ice cream samples, with average descriptor intensity values up to 25% higher. L80 3:1, L80 1:3, and L80 0:1 exhibited higher scores for attributes like “hardness,” “melting time” (time required for the product to melt when processed in the mouth), and “density/shape” (degree of compactness of the sample when pressed between the tongue and palate). Furthermore, L80 1:3 also had a higher intensity for the “sandy texture” descriptor, indicating a perceivable granular or non‐uniform consistency in the ice cream. Previously it was reported that greater substitution of fat in ice cream with a whey protein‐based substitute (Simplesse) resulted in a higher intensity in perceptions of the appearance (i.e., stickiness and air hole contents) and melting rate attributes of ice cream as assessed by a trained panel (Yilsay et al., 2006).

Also, for the 50% cream‐replaced ice creams, sample developed with only WPI showed a significantly higher intensity of the “hardness” descriptor, with an average score of 6.2, compared to 4.4 for the full‐fat ice cream (Figure 1a,aʹ). Hardness indicates the force required to compress the sample between the tongue and the palate. We found that the use of higher levels of WPI increased sensory hardness, and this increase was greater with higher levels of cream replacement. The increase in protein content of the ice cream through the addition of whey protein led to higher viscosity and reduced overrun. As a result, the ice creams were evaluated by the panelists as having texture descriptors that were significantly different from the control ice cream (Patel et al., 2006). With the increase in WPI in the ice cream, the sensory panelists reported a greater hardness and noted a faster meltdown in the samples containing 8% and 10% protein (Roy et al., 2022). This suggests that higher levels of protein increase the viscosity of the mix, which reduces its whipping ability and may affect the structure and incorporation of air into the ice cream, resulting in a denser and harder product.

The incorporation of WPI leads to a lower amount of water in the ice cream (Table 4), which can cause a higher hardness and less ductility since water exerts a plasticizing effect (Chuang & Yeh, 2006). Therefore, the lower water content increases ice cream hardness and brittleness (Jung et al., 2018). Moreover, Soukoulis et al. (2010) observed that reducing the milk fat content in ice cream, replacing it with hydrocolloids, is associated with higher sensory attributes of hardness, coarseness, and brittleness, supporting our findings. However, further analyses, such as determining the frozen/unfreezable water content through thermal analysis techniques such as DSC, would be necessary to better understand the relationship between the physical and sensory properties of low‐fat ice cream developed with the inclusion of aquafaba.

**TABLE 4:** Chemical composition, ΔE color and overrun of coffee ice cream samples.

	Chemical composition (wt%)							
Sample	Water	Carbohydrates	Lipids	Proteins	Fibers	kcal/100 g	ΔE color	Overrun %
Full‐fat	68	18.3	10.3	2.7	0.5	177		13
L50 1:0	73	18.3	5.3	2.6	0.5	132	2.07 ± 0.79	26
L50 3:1	70	18.3	5.3	5.5	0.5	135	3.25 ± 0.62	14
L50 1:1	67	18.3	5.3	8.5	0.5	147	3.64 ± 1.86	13
L50 1:3	64	18.3	5.3	11.4	0.5	159	4.22 ± 2.82	15
L50 0:1	61	18.3	5.3	14.4	0.5	180	5.04 ± 2.61	16
L80 1:0	76	18.3	2.3	2.6	0.5	105	4.14 ± 0.70	10
L80 3:1	71	18.3	2.3	7.3	0.5	115	4.60 ± 1.00	18
L80 1:1	67	18.3	2.3	12	0.5	134	6.92 ± 3.61	21
L80 1:3	62	18.3	2.3	16.7	0.5	153	5.99 ± 3.27	21
L80 0:1	57	18.3	2.3	21.4	0.5	172	5.17 ± 2.19	16

*Note*: See Table 1 for sample code details and formulations. ΔE values were calculated according to the formula provided in Section 2.4, comparing low‐fat samples with full‐fat ice cream.

As expected, the descriptor “ice crystal,” which indicates the perception of crystalline particles within the sample, showed the highest intensity in the L80 1:0 and L50 1:0 ice cream samples. This could be attributed to the higher amount of water provided by aquafaba combined with a reduced fat content. The presence of fat in the ice cream serves to inhibit the formation of ice crystals during the freezing process. It has been suggested that fat reduction implies an increase of ice crystals in low‐fat ice cream mixtures during freezing (Pintor‐Jardines et al., 2018), thereby contributing to a higher perception of ice crystal and harder texture in the resultant reduced‐fat ice cream product (Akbari et al., 2019). To address this issue, scientists have proposed solutions involving a blend of fat substitutes. These substitutes, including carbohydrate‐based options like inulin or modified starch, have been shown to slow down ice crystal growth by imparting texture properties similar to full‐fat ice cream (Akbari et al., 2016; Guinard et al., 1997; Roland et al., 1999). These results support our initial hypothesis that a combination of fat substitutes rather than a single ingredient could lead to a sensory perception of low‐fat ice cream that closely resembles that of full‐fat ice cream. In fact, our study showed that the use of aquafaba in combination with whey proteins resulted in slight differences in the sensory properties of fat‐reduced ice creams compared to the full‐fat ice cream, and the best combination was found with a 1:1 ratio of WAF to WPI (L50 1:1 and L80 1:1). Aquafaba imparts interesting foaming and emulsifying properties that can partially emulate cream in ice cream. However, excessive amounts of aquafaba can lead to structural collapse and an increased perception of ice crystals due to a reduction in total solids, negatively affecting the physical and sensory properties of the ice cream. On the other hand, whey proteins serve as a valuable source of protein that partially improves the viscosity and hardness of the mix, further supporting the structure and creaminess of the ice cream. The combined addition of WAF and WPI, leveraging the strengths of both ingredients, facilitated the development of a low‐fat ice cream that exhibited fewer differences compared to its full‐fat counterpart.

Ice creams L80 1:0, 3:1, 1:1, 1:3, and 0:1, exhibited increased scores in the brown color descriptor (Figure 1aʹ). This effect can be attributed to the decreased contribution of white color from the cream, as found by Yilsay et al. (2006), in which the sensory panel rated the ice cream samples as less white as milk fat content decreased. At 50% cream replacement, the increase in brown color was not perceived by the panelists. Similar results have also been reported in low‐fat chocolate ice cream (Prindiville et al., 2000).

Regarding the taste and aroma descriptors, compared to control full‐fat ice cream, the L50 1:3 and L50 1:1 samples had a higher intensity of “coffee” aroma descriptor, and the L50 1:1 sample had a lower intensity of the “sweetness” descriptor, while the L50 0:1 sample received higher scores for “bitterness” taste descriptor (Figure 1b). Soukoulis et al. (2010) found that the milk fat content contributes to higher aroma and sweetness attributes while reducing bitterness. As expected, in ice creams where 80% of the cream has been replaced, these differences in taste and aroma descriptors are more pronounced (Figure 1bʹ), highlighting the crucial role of fat as a modulator of flavor perception.

### 3.2 Physical analyses

Overrun, melting rate, texture, and color properties of the experimental low‐fat ice creams were measured and compared with those of the full‐fat ice cream counterpart to evaluate the physical characteristics of the low‐fat ice cream developed with the use of WAF. Table 4 presents, in addition to the overrun value, the ΔE color values calculated based on the CIELAB color values of the full‐fat sample compared to the low‐fat samples, the chemical composition, and the caloric content of the ice creams calculated in relation to the ingredients used for the different ice cream formulations. The replacement of cream with aquafaba helps reduce the calorie intake, as its contribution on the caloric content of the ice cream is insignificant. Therefore, the ice creams developed with the replacement of 50% and 80% of the cream with only WAF (L50 1:0 and L80 1:0) have reductions of 25% and 40% in calorie content, respectively, compared to the full‐fat sample. Overall, a higher fat replacement resulted in a greater color difference compared to the full‐fat ice cream (Table 4). This was also observed in the sensory analysis (Figure 1), where a 50% cream replacement with fat replacers was not assessed as significantly different by the panelists. In contrast, the 80% replacement led to significant differences in sensory perception. Aquafaba also increased the overrun, that is, the amount of air incorporated into the ice cream, due to its foaming ability (Erem, Akdeniz et al., 2023; Mustafa et al., 2018). The full‐fat control sample had an overrun of 13%, while in the experimental samples the overrun ranged from 10% to 26%. This result is in line with the overrun values measured in ice creams produced with a batch‐type freezing process (Akalın et al., 2008). L50 1:0, in which half of the cream content in the ice cream was replaced by WAF, had the highest overrun value of 26%, highlighting the effective foaming properties of aquafaba in enhancing the volume of the ice cream (Erem, Akdeniz et al., 2023). Overall, the 50% cream replacement caused an increase or similar values of overrun to the control sample in ice creams (Table 4). These results are in agreement with Javidi and Razavi (2018), which reported that partially reducing fat in ice cream can lead to an increase in overrun values. However, Yilsay et al. (2006) reported a slight decrease in overrun values in low‐fat (50% replacement) and fat‐free (100% replacement) ice creams in which the fat was replaced with a whey protein substitute. In our study, the replacement of the cream with WPI (samples denoted as L50 0:1 and L80 0:1) did not cause a substantial difference in overrun values compared to full‐fat ice cream. However, it is worth noting that the functionality of whey protein depends on the type of ingredient used. Contrary to our study, Yilsay et al. (2006) used microparticulated and thermodenatured whey protein (Simplesse). Protein denaturation by thermal or mechanical treatments can affect its structure and functional properties, hence providing different foaming and gelling capacities depending on the type of whey protein used (Balivo, d'Errico, et al., 2024; Nunes & Tavares, 2019).

The replacement of 80% cream with WAF (L80 1:0) did not cause an overrun increase as observed in the 50% replacement; instead, it results in a slight decrease. The greater disruption of the fat globule network resulting from the increased substitution of milk fat has an impact on the structure of the ice cream (Soukoulis et al., 2010; Yilsay et al., 2006), causing the air bubbles to collapse and consequently reducing the overrun. In fact, Karaca et al. (2009) noted that partial fat replacement in ice cream exhibited the highest degree of overrun. On the contrary, when they totally replaced the fat from the ice cream, the overrun was less than the control full‐fat sample.

**FIGURE 2:**
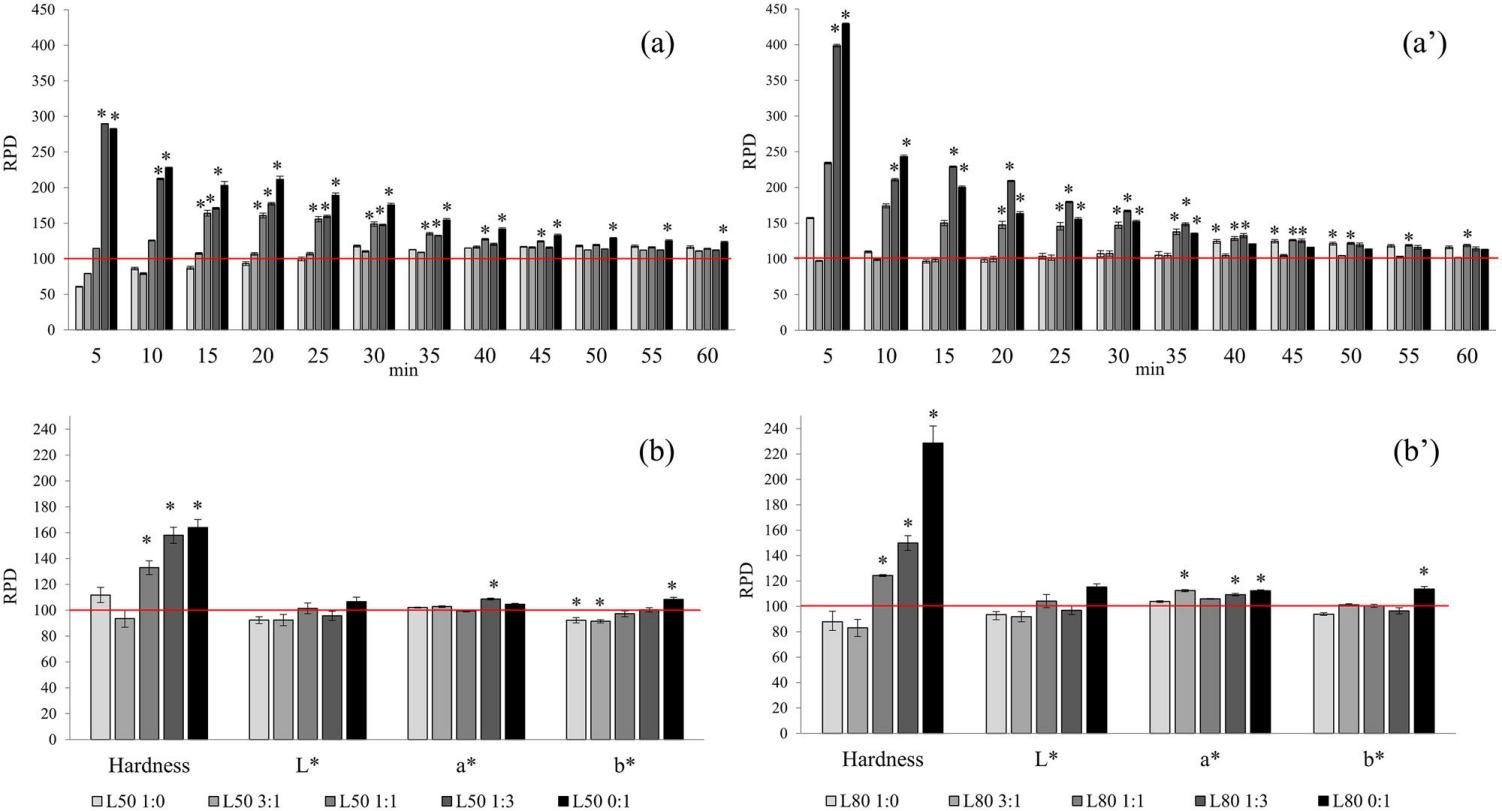
Melting rate (a and aʹ), hardness, and L*, a*, and b* color values (b and bʹ) evaluated in 50% and 80% cream replacement ice cream samples. Values were reported as a relative percentage difference (RPD) (increase or decrease) compared to the full‐fat ice cream set at 100%. The asterisk indicates significant differences (*p* < 0.05) compared to the full‐fat control ice cream. See Table 1 for sample code details.

Figure 2 illustrates the results of melting rate, texture, and color analysis, expressed as a percentage change relative to the full‐fat control ice cream set at 100% (horizontal line). There is a clear trend of increase in the melting rate of the ice cream as the amount of cream replaced increases (50% and 80%, respectively, Figure 2a,aʹ) and as the amount of WPI increases. The presence of fat slows the rates of heat transfer through full‐fat ice cream (Genovese et al., 2022), resulting in slower melting. Combining WPI with aquafaba in cream replacement reduces the melting rate of experimental ice creams to levels similar to full‐fat ice cream (e.g., for L50 3:1, L50 1:1, and L80 3:1 samples), especially when considering a reasonable consumption time of approximately 15 min for the ice cream.

A similar behavior is exhibited by hardness, which increased both with increasing the amount of cream replaced and with increasing WPI as substitutes for cream in ice cream (Figure 2b,bʹ). Particularly, the L50 1:1, L50 1:3, L50 0:1, L80 1:1, L80 1:3, and L80 0:1 samples had a higher hardness value (*p* < 0.05) than full‐fat ice cream. The greatest hardness was found in the samples where the cream was totally replaced by WPI, in agreement with the results of the sensory analysis (Figure 1). The L50 0:1 and L80 0:1 samples had approximately 64% and 129% higher hardness, respectively, than full‐fat ice cream. Conversely, the higher hardness of the samples L50 1:1 (33%) and L80 1:1 (24%) samples compared to full‐fat ice cream was not perceived by the sensory panel. In accordance with the present results, Akalın et al. (2008) observed an increase in hardness in low‐fat (40% replacement of milk fat) and fat‐free (70% replacement of milk fat) ice creams developed with WPI. Specifically, ice creams incorporating WPI exhibited the highest level of hardness, surpassing that of full‐fat ice cream by over 200%. The authors also replaced the cream with inulin, still obtaining an increase in hardness compared to the control sample, although less than that caused by the use of WPI. In the Figure 2, hardness was reported as a percentage increase in relation to full‐fat ice cream placed at 100%. However, hardness values, reported in Table S1, ranged from 17.04 ± 1.90 N (full fat) to 38.95 ± 13.45 N (L80 0:1). Table S1 also presents the instrumental color values in CIELAB coordinates.

We observed that the ice creams formulated with WPI had generally lower overrun values, resulting in greater hardness. This aligns with the findings of Muse and Hartel (2004), who developed statistical models to study the structural elements of ice cream affecting melting rate and hardness. Increasing the protein content by adding WPI led to higher ice cream mix viscosity, loss and storage modulus, and a reduction in overrun (Roy et al., 2022). Additionally, instrumental measurements showed that both the hardness and melting rate of the ice cream increased with WPI addition (Roy et al., 2022).

Therefore, this increase in hardness could be directly related to the rheological properties, that is, the higher apparent viscosity (consistency coefficient) that WPI provides to low‐fat ice cream, changing its flow properties with greater variation from a Newtonian fluid than that of full‐fat ice cream (Akalın et al., 2008; Muse & Hartel, 2004). The higher intensity of the “density/shape” descriptor for L50 0:1 and L80 0:1 samples (Figure 1a,aʹ) supports this hypothesis, as milk proteins can bind to water and create networks that trap water molecules, resulting in a denser structure and greater resistance to deformation. In our study, aquafaba, especially in combination with WPI, helped to mimic physical properties of full‐fat ice cream, supporting the hypothesis of the use of combinations of fat replacers to develop ice creams with sensory properties similar to full‐fat ones.

The higher value (*p* < 0.05) for the color parameters a* (red) and b* (green/yellow), for example, for samples L50 1:3, L50 0:1, L80 1:3, and L80 0:1 (Figure 2b,bʹ), suggests a higher intensity of the brown color as perceived by the panelists in the sensory analysis. The greater intensity of the brown color, brought by the coffee, is also due to the consequent reduction of the amount of cream, characterized by its white color (Yilsay et al., 2006), in low‐fat ice creams.

### 3.3 Volatile compounds analysis

A total of 14 volatile compounds were monitored in the dynamic headspace of ice cream samples; 13 of them are reported as key odors of coffee (Ayseli et al., 2021). They are 7 pyrazines, 2 aldehydes, 2 ketones, and 2 volatile phenols. On the contrary, 2‐acetylthiazole is identified as the most volatile compound in aquafaba (Table 3). Table 3 also shows the odor threshold values calculated in water, the log P_ow_, and the odor descriptors taken from the literature.

**FIGURE 3:**
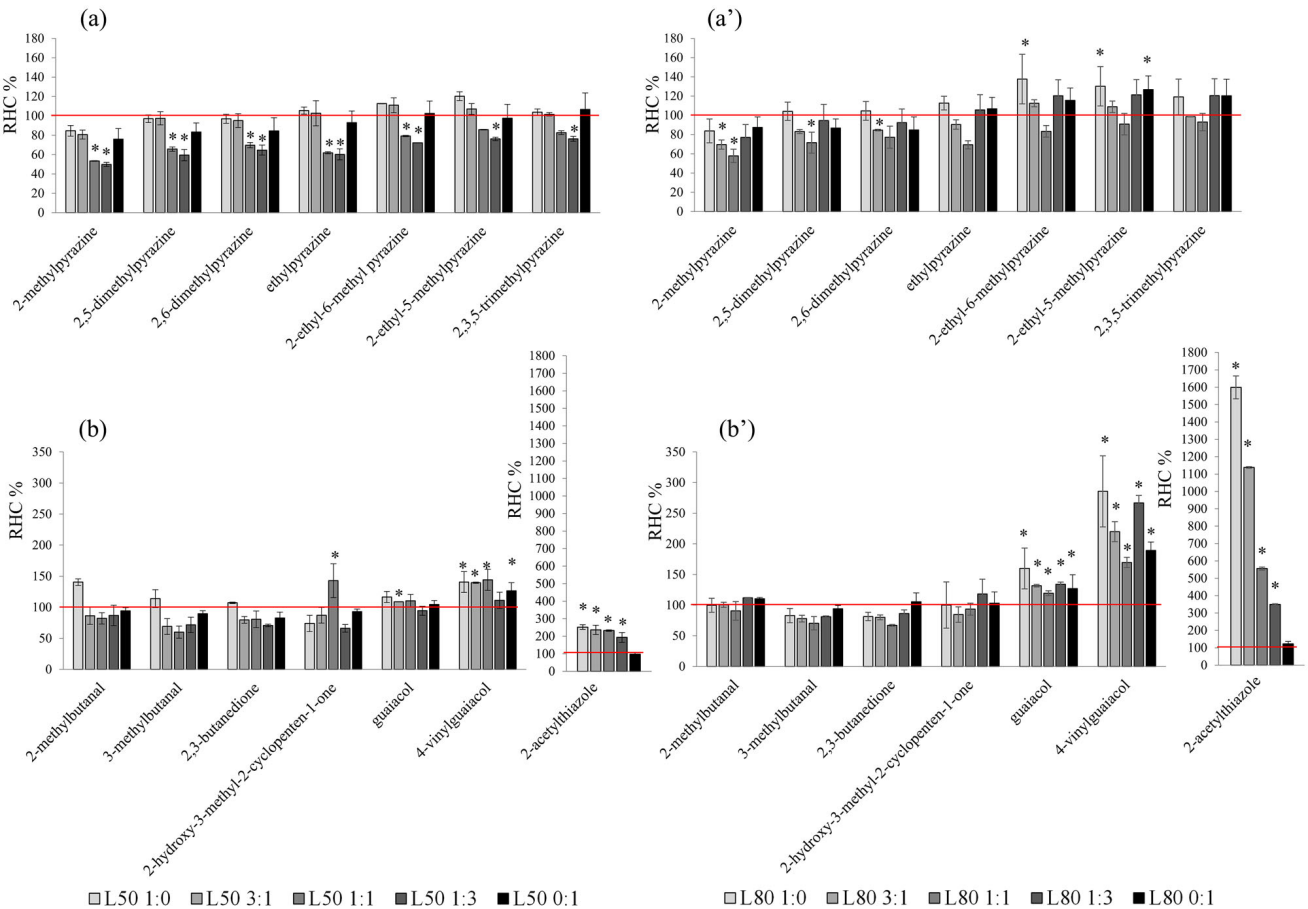
Level of pyrazines (a and aʹ), aldehydes, ketones, volatile phenols, and 2‐acetylthiazole (b and bʹ) released in the headspace of 50% and 80% cream replacement ice cream samples. The values have been reported as relative headspace concentration (RHC) compared to the full‐fat ice cream set at 100% (horizontal line). The asterisk indicates significant differences (*p* < 0.05) compared to the full‐fat control ice cream. See Table 1 for sample code details.

Figure 3 shows the levels of pyrazines, aldehydes, ketones, volatile phenols, and 2‐acetylthiazole released in the headspace of L50 and L80 ice cream samples. The values are given as relative headspace concentrations compared to the full‐fat ice cream, which was set at 100%. A value <100% indicates that the volatile compound had a lower release in the headspace of the ice cream sample and a higher retention by the ice cream matrix. This is an indication of a more gradual release in the mouth, which prolongs aroma persistence during ice cream consumption. On the contrary, a value higher than 100% indicates that the volatile compound showed a higher release in the headspace of the ice cream sample, indicating a lower retention and persistence during its consumption.

As expected, 2‐acetylthiazole resulted in higher levels with the increasing of WAF in the ice cream (Figure 3). It is characterized by a low odor threshold with popcorn and roasted odors (Table 3). However, according to the sensory analysis, this note was not perceived in the coffee ice cream (Figure 1). This is probably because it was masked by the coffee aroma. The ice cream with higher fat substitution showed a higher release of volatile compounds in the headspace. Furthermore, it is interesting to note that the ice creams in which cream was replaced with a combination of WAF and WPI showed higher retention of pyrazines. In particular, the release of pyrazines in the headspace was similar between L50 3:1 and the control full‐fat sample. This similarity in pyrazine release contributed to a comparable perception of coffee aroma, as determined in the sensory analysis (Figure 1b,bʹ). In contrast, the release of pyrazines in the L50 1:1 and L50 1:3 samples was significantly lower than in the control sample (Figure 3a,aʹ), suggesting a more gradual release in the mouth during consumption and a more persistent perception of the coffee aroma, as evidenced by the results of the sensory analysis (Figure 1b,bʹ).

In the samples in which the cream was completely replaced with WPI, the total water content in the ice creams was lower (Table 4), implying an increase in the concentration of pyrazines in the headspace (Figure 3). Previously, Welty et al. (2001) found that the concentration of most selected volatile compounds increased with decreasing fat concentration in ice cream, and whey protein‐based fat replacers (such as Simplesse and Dairy‐Lo) generally increased the concentration of volatiles compared to milk fat or cocoa butter. In particular, the total peak area of some pyrazines in chocolate ice cream, that is, 2‐ethyl‐3‐methyl pyrazine, 2‐ethyl‐3,5‐dimethyl pyrazine, tetramethyl pyrazine, and isobutyl pyrazine, increased when milk fat was replaced, suggesting less retention in low‐fat ice creams (Welty et al., 2001).

Pyrazines exhibit a relatively low log P_ow_, ranging from 0.49 to 1.58 (Table 3), indicating their low hydrophobicity. In fact, the addition of aquafaba leads to an increased total water content in the ice cream, causing a decreased release of pyrazines into the headspace, enhancing the persistency of the sensory coffee aroma perception, rated as having higher intensity in the QDA (Figure 1b,bʹ). The L50 1:1 and L50 1:3 samples exhibited a lower level of pyrazine release compared to both the control and the other samples. This behavior can be attributed to the simultaneous presence of aquafaba, which increases the affinity of pyrazines with the ice cream matrix due to higher water content, and WPI, which might establish weak bonds with pyrazines (Ma et al., 2019), limiting their release into the sample headspace. This could result in a prolonged persistence of the aroma during ice cream consumption, which is appreciated by the consumer of ice cream (Genovese et al., 2022). Conversely, the release of guaiacol and 4‐vinylguaiacol, which are responsible of smoky aroma, was higher in comparison to the full‐fat control sample. Among the compounds responsible for the coffee aroma, 4‐vinyl guaiacol is the most hydrophobic (with a log P_ow_ of 2.24, Table 3).

As expected, in the L80 samples, where 80% of the cream has been replaced, the distribution of all volatile compounds in the headspace increased as the fat content of the ice cream decreased. This was particularly observed for phenolic compounds, which have the highest log P_ow_ among the volatile compounds monitored (Table 3). In fact, guaiacol and 4‐vinylguaiacol exhibited higher release in all L80 ice cream formulations compared to the full‐fat ice cream (Figure 3). Some pyrazines, such as 2‐ethyl‐6‐methyl pyrazine and 2‐ethyl‐5‐methyl pyrazine, showed higher release in the L80 1:0 sample, while 2‐methylpyrazine, 2,5‐ and 2,6‐dimethylpyrazine showed lower release, particularly in L80 1:1 (1:1 WAF:WPI ratio) ice cream formulations. Since fat plays a crucial role as a solvent for the retention of aroma compounds (Feyzi et al., 2020), our results suggest that the different release of coffee volatile compounds in low‐fat ice cream made with 80% of cream replacement could lead to slight differences in the nuances and persistence of the coffee aroma, from roasted and nutty, typical of pyrazines, to burnt and smoked, typical of volatile phenols (Table 3).

**FIGURE 4:**
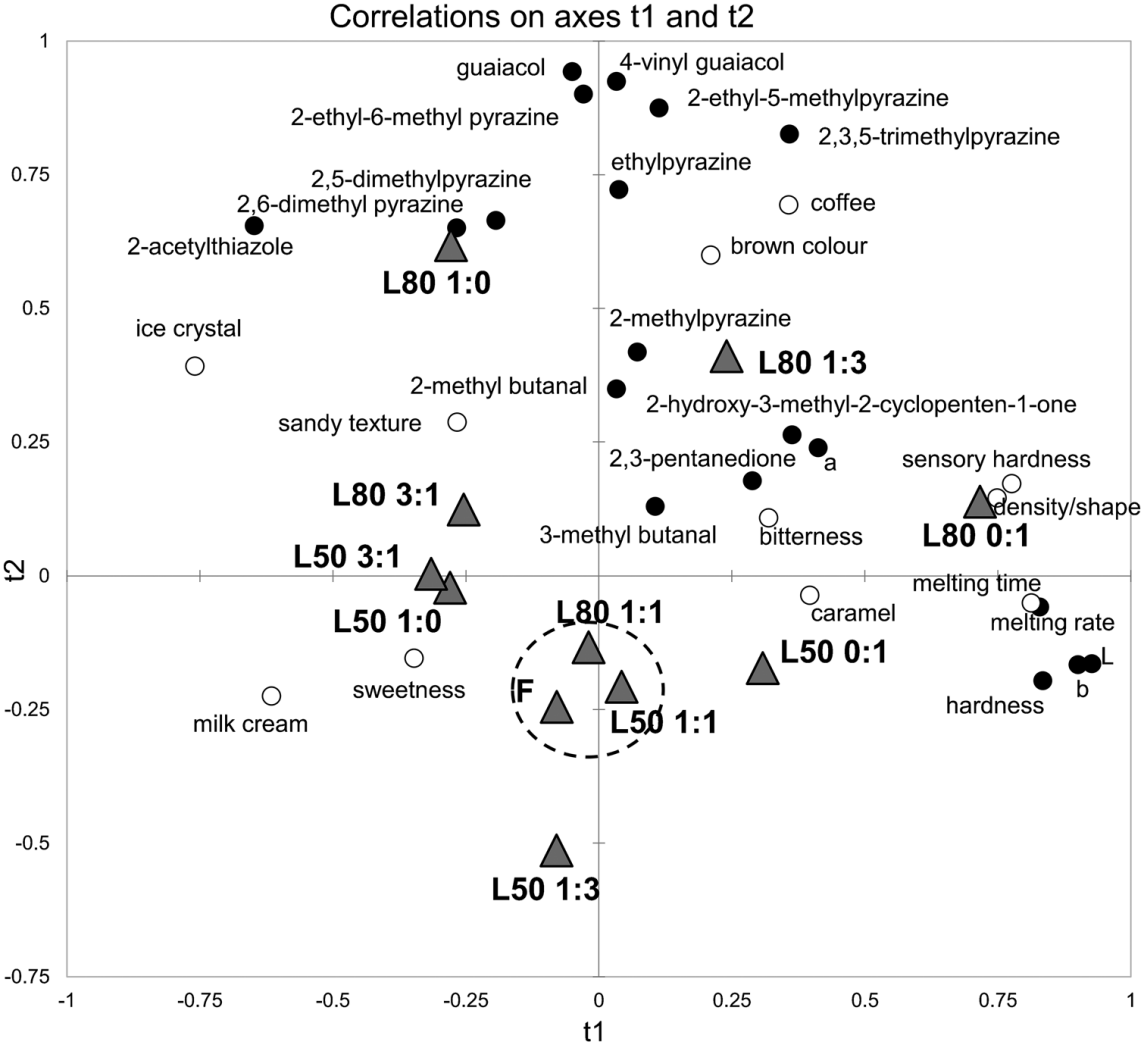
Partial least squares regression (PLSR) loading plot (t1 vs. t2) for instrumental data (volatile organic compounds, hardness, color, and melting rate) (filled circles) and sensory descriptors (unfilled circles) of 50% and 80% cream replacement ice cream samples. See Table 1 for sample code details.

Partial least square regression (PLSR) analysis was conducted to investigate the relationships between instrumental data (volatile compounds, melting rate, hardness, and color) and sensory attributes of the ice cream samples (Figure 4). The instrumental data were selected as the *X* variables and the sensory response values as *Y* variables. As shown in the PLSR plot, a good agreement between the instrumentally measured data and those obtained in the sensory analysis was achieved.

As expected, the descriptor “milk cream” characterized the full‐fat control ice cream. Pyrazines, guaiacol, 4‐vinylguaiacol, 2 and 3‐methylbutanal showed a good correlation with the descriptor “coffee.” Moreover, as mentioned above, ice cream with 80% cream replacement tends to have a higher headspace release of coffee volatile compounds but a lower aroma persistence during its consumption, especially when the fat has been replaced by a single replacer. For example, the L80 1:0 sample, in which 80% of the cream was replaced with WAF only, was positively associated with the sensory descriptor “ice crystal” and had a more intense brown color and a greater amount of coffee and volatile compounds in the headspace, such as pyrazines, volatile phenols, and 2‐acetylthiazole. On the opposite side of the plot, L80 0:1, where 80% of the cream was replaced by WPI, showed the highest instrumental hardness and fastest melting rate, aligning with the corresponding sensory attributes. The PLSR plot clearly indicates a high degree of similarity between the ice creams where 50% and 80% of the cream was replaced with a 1:1 combination of aquafaba and whey proteins (L50 1:1 and L80 1:1) and the control ice cream (F). The physical and structural attributes of ice cream, as observed by Chung et al. (2004) in their study on low‐fat ice cream samples, play a crucial role in discriminating the samples. Changes in texture resulting from fat replacement can also influence the release and perception of ice cream aroma (Gierczynski et al., 2011).

Hence, we conducted a triangle test between sample F and sample L50 1:1, and between sample F and L80 1:1, to test whether the panel could correctly identify the low‐fat ice creams that were found to be most similar to full‐fat ice cream. Sample L50 1:1 was correctly identified 7 times out of 12, while L80 1:1 was recognized 9 times out of 12. While the results for the comparison between F and L50 1:1 were not significant, the sample L80 1:1 was discriminated from *F* (*α* = 0.05). Therefore, in this study, the ice cream in which 50% of the cream was replaced with a 1:1 blend of aquafaba and WPI did not prove to be perceived as different from the full‐fat one.

## 4 CONCLUSION

This study highlights the importance of using a combination of fat substitutes to achieve sensory characteristics and physical properties more similar to those of full‐fat ice cream. In fact, these findings support our initial hypothesis and establish aquafaba as a potential replacer for cream in ice cream, especially when combined with WPI.

Combining aquafaba and WPI in a 1:1 ratio to replace 50% of milk cream resulted in ice cream with sensory and physical properties very similar to those of full‐fat ice cream. This sample was not identified as different from the full‐fat counterpart in the triangle test. Overall, other WAF:WPI combinations examined in this study also produced ice creams with satisfactory sensory and chemical properties, which may appeal to a consumer by virtue of a nutritional benefit of the ice cream, such as reduced calorie or increased protein content.

However, replacing 80% rather than 50% of the cream from the ice cream caused greater differences in the sensory attributes of appearance, texture, and aroma, as well as instrumental hardness and melting rate. The replacement of milk cream with WPI alone showed the most significant differences compared to the full‐fat ice cream. These differences were mainly in texture and physical properties. On the other hand, replacing cream solely with aquafaba resulted in a higher perception of ice crystals and a faster release of volatile compounds, indicating shorter aroma persistence in the mouth during ice cream consumption. In contrast, replacing cream with a combination of aquafaba and WPI led to a more controlled release of coffee aroma, as observed through both instrumental and sensory analyses.

Further research should explore novel approaches for the synergistic use of fat replacer ingredients, with a focus on identifying optimal combinations that preserve the sensory and physical properties of low‐fat ice creams. Furthermore, in‐depth studies on the incorporation of vegetable by‐products could help to develop sustainable and economically efficient strategies, thus promoting circular economy principles in the low‐fat dairy product industry.

## AUTHOR CONTRIBUTIONS

**Andrea Balivo**: Conceptualization; investigation; formal analysis; visualization; writing—original draft; data curation; methodology. **Giulia d'Errico**: Writing—original draft; formal analysis; visualization; investigation. **Alessandro Genovese**: Conceptualization; writing—review and editing; supervision; resources; investigation; formal analysis; methodology; data curation.

## CONFLICT OF INTEREST STATEMENT

The authors declare no conflicts of interest

## Supporting information




**Table S1**. CIELAB colour parameters (L*, a*, b*) and hardness values (in Newtons) for ice cream samples with varying levels of cream replacement.

## Data Availability

Data will be made available on request.
