# Supplementary material for: Whipped chickpea aquafaba as a fat replacer in ice cream: Effect on sensory and physicochemical properties
Source: J Food Sci. 2024 Dec 15;89(12):8730–45. doi: 10.1111/1750-3841.17605 (PMC11673448; doi:10.1111/1750-3841.17605)
Supplement: Supplementary file 1 — Table S1. CIELAB colour parameters (L*, a*, b*) and hardness values (in Newtons) for ice cream samples with varying levels of cream replacement. [file JFDS-89-8730-s001.docx]

**Table S1.** CIELAB colour parameters (L*, a*, b*) and hardness values (in Newtons) for ice cream samples with varying levels of cream replacement.

| **Sample** | **L* (lightness)** | **a* (red-green index)** | **b* (yellow-blue index)** | **Hardness (N)** |
| --- | --- | --- | --- | --- |
| Full-fat | 38.6 ± 0.59 | 9.84 ± 0.31 | 20.51 ± 0.93 | 17.04 ± 1.90 |
| L50 1:0 | 40.07 ± 0.79 | 9.71 ± 0.29 | 20.95 ± 0.67 | 19.04 ± 5.71 |
| L50 3:1 | 40.69 ± 1.23 | 9.72 ± 0.28 | 19.56 ± 2.27 | 15.93 ± 5.87 |
| L50 1:1 | 41.44 ± 2.08 | 9.49 ± 0.64 | 20.85 ± 1.16 | 22.64 ± 6.67 |
| L50 1:3 | 41.66 ± 2.50 | 10.44 ± 0.79 | 22.53 ± 1.88 | 26.90 ± 5.37 |
| L50 0:1 | 48.45 ± 3.55 | 9.79 ± 1.63 | 23.57 ± 2.97 | 27.93 ± 6.24 |
| L80 1:0 | 42.60 ± 0.88 | 9.73 ± 0.78 | 20.77 ± 1.44 | 14.97 ± 8.29 |
| L80 3:1 | 43.80 ± 1.23 | 9.79 ± 0.48 | 19.56 ± 2.27 | 14.15 ± 6.76 |
| L80 1:1 | 45.24 ± 2.89 | 9.91 ± 0.33 | 22.00 ± 1.32 | 21.18 ± 2.69 |
| L80 1:3 | 44.00 ± 3.27 | 10.22 ± 0.95 | 20.99 ± 2.48 | 25.55 ± 5.76 |
| L80 0:1 | 52.35 ± 2.64 | 10.50 ± 0.72 | 24.71 ± 2.15 | 38.95 ± 13.45 |

Data are presented as mean ± standard deviation.
